# Up-regulation of microRNA-135 or silencing of PCSK6 attenuates inflammatory response in preeclampsia by restricting NLRP3 inflammasome

**DOI:** 10.1186/s10020-021-00335-x

**Published:** 2021-07-23

**Authors:** Xiaolan Zhao, Xun Zhang, Zhao Wu, Jie Mei, Lingling Li, Yujue Wang

**Affiliations:** Genaecology and Obstetrics Department, Sichuan Academy of Medical Sciences, Sichuan Provincial People’s Hospital, No. 32, West Second Section First Ring Rd, Chengdu, 610072 China

**Keywords:** Preeclampsia, MicroRNA-135, PCSK6, NLR pyrin domain containing 3

## Abstract

**Objective:**

Numerous studies have confirmed the correlation of microRNAs (miRNAs) with human disease, yet few have explored the role of miR-135 in preeclampsia (PE). This study intends to discuss miR-135’s function in inflammatory response in PE by modulating proprotein convertase subtilisin/kexin-6 (PCSK6) and NLR pyrin domain containing 3 (NLRP3).

**Methods:**

The venous blood and placental tissues were collected from PE pregnant women and 25 normal ones. The levels of miR-135, PCSK6 and NLRP3 in placenta tissues of patients were detected. Hypoxia/reoxygenation HTR-8/SVneo and HPT-8 models were established to mimic PE in vitro, and cell proliferation, colony formation, apoptosis rate, invasion, migration and inflammation were detected through gain-of and loss-of-function assays.

**Results:**

MiR-135 was down-regulated, and PCSK6 and NLRP3 were up-regulated in PE patients. Up-regulating miR-135 or silencing PCSK6 strengthened colony formation ability, viability, invasion and migration ability, and weakened apoptosis and inflammation of H/R-treated HTR-8/SVneo and HPT-8 cells. Inhibition of NLRP3 negated the effects of silenced PCSK6 in H/R-treated HTR-8/SVneo and HPT-8 cells.

**Conclusions:**

Altogether, we demonstrate that up-regulated miR-135 or reduced PCSK6 attenuates inflammatory response in PE by restricting NLRP3 inflammasome, which provides novel therapy for PE treatment.

## Introduction

Preeclampsia (PE) refers to a pregnancy-specific syndrome which influences 3–5 % of pregnancies and usually appears when a woman presents hypertension and proteinuria after about 20 weeks of gestation (Mol et al. 2016; Sircar et al. 2015). It is generally acknowledged that the physiopathological process of PE starts with deficient trophoblast invasion during the early period of pregnancy, which leads to a rise in oxidative stress and promotes systemic endothelial dysfunction in the later stages of PE, resulting in the distinctive PE clinical manifestation (Correa et al. 2016). The diagnostic criteria for PE include the presence of Hypertension and at least one of the following emerging diseases, including maternal end organs or uterine placental dysfunction (Dymara-Konopka et al. 2018). Patients with severe PE may experience headaches, visual disturbances, upper abdominal pain or nausea and vomiting (Mol et al. 2016). Treatment of PE is difficult because the progress of an established PE can’t be totally reversed or stopped (El-Sayed 2017). Therefore, we conduct this study to grope for more effective approaches to PE treatment.

MicroRNA (miRNAs), a collection of small, endogenous non-coding RNAs with approximately 21–24 nucleotides, have the capacity to modulate gene expression by regulating mRNAs’s stability, govern the translation rates, and therefore modulate protein synthesis (Zaheer et al. 2019). MiR-135, a significant member of miRNA cluster, has been documented to suppress glycolysis in pancreatic ductal adenocarcinoma and regulate proliferation and epithelial-mesenchymal transition in breast cancer (Yang et al. 2019; Jiang et al. 2019). Evidence has proved that reduced miR-126-3p expression gives rise to incremental inflammatory response in PE (Xu et al. 2019). Zhang et al. have demonstrated that miR-942 expression falls prior to 20 weeks gestation in PE women and is related with the pathophysiology of PE in vitro (Zhang et al. 2017a, b).

It is suggested that miR-124 depresses prostrate cancer cell multiplication and aggression via proprotein convertase (PC) subtilisin/kexin-6 (PCSK6, also named PACE4) pathway (Kang et al. 2014). PCSK6 is a member of the PC family of serine endoproteases within the cell secretory pathway (Panet et al. 2017). PCSK6 has been confirmed as candidate gene of glioma cell invasion, which can be circumscribed by abatement of PCSK6 (Delic et al. 2012). LR pyrin domain containing 3 (NLRP3) is a well-known member of inflammasomes that are intracellular complexes associated with the innate immunity and transform prointerleukin-1β (proIL-1β) and proIL-18 into mature forms and start pyroptosis by cleaving procaspase-1 (Hoseini et al. 2018). NLRP3 has been regarded as the guard of cellular integrity and its activation is fundamentally important in a plethora of aseptic and pathogen-triggered inflammatory environment (Gaidt and Hornung 2018). Specially, NLRP3 inflammasome is involved in pregnancy dysfunction, including PE (Shirasuna et al. 2020) and higher NLRP3 inflammasome is related to exaggerated inflammatory state in PE (Weel et al. 2017). Therefore, in this study, we mean to examine the impacts of miR-135 on the inflammatory response in PE by modulating PCSK6 and NLRP3 inflammasome.

## Materials and methods

### Ethics statement

All participants signed a document of informed consent. The protocols of this study were approved by the Ethic Committee of Sichuan Academy of Medical Sciences & Sichuan Provincial People’s Hospital (ethical number: 201600618) and based on the ethical principles for medical research involving human subjects of the Helsinki Declaration.

### Study subjects

Twenty-five pregnant women with PE who were hospitalized and delivered in the obstetrical department in Sichuan Academy of Medical Sciences & Sichuan Provincial People’s Hospital from January 2017 to September 2018 (PE group) were included in this study, with the average age of 28.91 ± 5.42 years and average gestational week of 37.03 ± 0.92 weeks. Twenty-five normal pregnant women in the third trimester of pregnancy were selected as control group, with the average age of 26.73 ± 4.34 years and average gestational week of 37.31 ± 0.59 weeks. Patients in both groups all had no history of chronic hypertension, diabetes, infection, nephritis and systemic lupus erythematosus, and no other pregnancy complications.

### Specimen collection

After all study subjects fasted for 8–12 h, 5 mL cubital venous blood was taken, followed by serum separation and storage at −70 °C. Within 30 min after delivery of the placenta, placenta tissues were taken from the center of the placenta (avoiding necrotic and calcified regions) for paraffin section preparation and total RNA and protein extraction.

### Hematoxylin-eosin (HE) staining

The specimens were cut into 4-µm sections and baked after 10 % formaldehyde fixation and paraffin embedment. Then tissue sections were sequentially dewaxed in xylene I and xylene II for 10 min each time, and successively dehydrated in absolute ethanol I, absolute ethanol II, 95 % alcohol, 80 % alcohol and 70 % alcohol (2 min each). Then the sections were stained with hematoxylin, differentiated with 1 % hydrochloric acid alcohol and then successively immersed in 50 %, 70 %, and 80 % alcohol (2 min each). After soaked in eosin for 5 s, the sections were successively immersed into 95 % alcohol, absolute ethanol I and absolute ethanol II again (3 min each), and then in xylene I and xylene II (5 min each), followed by sealing with neutral gum and microscopic examination.

### Enzyme linked immunosorbent assay (ELISA)

Serum samples were centrifuged for supernatant collection. The cell culture supernatants were placed into sterile eppendorf tubes. Eight standards were prepared based on the specifications of IL-1β and TNF-α ELISA kit (Raybiotech, Georgia, USA), with the eighth well as the blank control. Then 100 µL prepared standard was added to the 96-well plate which was then sealed with a membrane for 2-h incubation at 37 °C. Then each well was supplemented with 100 µL primary antibody incubated for 1 h and mixed with 100 µL prepared secondary antibody and shook gently for 45 min at 37 °C. Subsequently, 100 µL color developer was added to each well and incubated for 0.5 h in the dark, and then 50 µL stop solution was add to stop the reaction. The optical density (OD) value and concentration of each well was measured immediately, and the standard curve was drawn.

### Terminal deoxyribonucleotidyl transferase-mediated deoxyuridine triphosphate-digoxigenin nick end labeling (TUNEL) staining

The paraffin-embedded sections were routinely dehydrated, dehydrated and rinsed, followed by 15-min incubation at 37 °C with proteinase K. Then 50 µL TUNEL reaction mixture (ZSGB-Bio, Beijing, China) was added dropwise and incubated in a humid box at 37 °C for 60 min. Next, 50 µL Converter-POD was added and incubated at 37 °C for 30 min, followed by adding diaminobenzidine color developer and observation under the microscope. Subsequently, the sections were stained with hematoxylin for 2 min, dipped in 95 % ethanol I–II, and immersed in anhydrous ethanol I–II (3–5 min each) and xylene I–II (3–5 min each), followed by sealing with neutral gum. Finally, the trophoblast apoptosis index was calculated under the light microscope. All operations were performed by double-blind method.

### Quantitative polymerase chain reaction (qPCR)

Total RNA in tissues and cells was extracted with a Trizol extraction kit (Invitrogen, California, USA), and the concentration and purity of the RNA were determined using nanodrop2000 micro ultraviolet spectrophotometer (1011U, nanodrop, USA). PCSK6 and NLRP3 were detected by reverse transcription kit (RR047A, Takara, Japan) to obtain cDNA according to the protocol of TaqMan MicroRNA Assays Reverse Transcription primer (4427975, Applied Biosystems, USA). ABI7500 quantitative PCR instrument (7500, ABI, USA) was utilized for real-time fluorescent quantitative PCR. U6 and glyceraldehyde-3-phosphate dehydrogenase (GAPDH) were the internal reference. The relative transcription level of miR-135, PCSK6, and NLRP3 was calculated by 2^−ΔΔCt^ method (Ayuk et al. 2016). Table 1 shows the primers.

**Table 1:** Primer sequence

Gene	Primer sequence (5′–3′)	GenBank number
miR-135	Forward: 5′-GCTTATGGCTTTTCATTCCT-3′	NR_029893.1
	Reverse: 5′-GTGCAGGGTCCGAGGT-3′	
U6	Forward: 5′-CTCGCTTCGGCAGCACATATACTA-3′	NR_002752.2
	Reverse: 5′-ACGAATTTGCGTGTCATCCTTGCG-3′	
PCSK6	Forward: 5′-ACTCCAGAAGAAGAGGAAGAGTA-3′	NM_138319.4
	Reverse: 5′-ACCATCGCAGCCTTTATCA-3′	
NLRP3	Forward: 5′-CCAGGGCTCTGTTCATTG-3′	NM_001191642.1
	Reverse: 5′-CCTTGGCTTTCACTTCG-3′	
Bcl‐2	Forward: 5′‐ATGTGTGTGGAGAGCGTCAA‐3′	NM_000633.2
	Reverse: 5′-ACAGTTCCACAAAGGCATCC-3′	
Bax	Forward: 5′‐GGGGACGAACTGGACAGTAA‐3	NM_004324.3
	Reverse: 5′-CAGTTGAAGTTGCCGTCAGA-3′	
GAPDH	Forward: 5′-TTGTGTCCGTCGTGGATCTGA-3′	NM_008084.2
	Reverse: 5′-TTGCTGTTGAAGTCGCAGGAG-3′	

*miR-135* microRNA-135, *PCSK6* proprotein convertase subtilisin/kexin-6, *NLRP3* NLR pyrin domain containing 3, *Bcl-2* B cell lymphoma 2, *Bax* Bcl-2-associated X, *GAPDH* glyceraldehyde-3-phosphate dehydrogenase

### Western blot analysis

Total protein in placental tissues and cells was extracted through radio-immunoprecipitation assay lysis buffer (Solarbio, Beijing, China) and quantified by BCA kit (P0012; Beyotime, Shanghai, China). The protein (50 µg) was dissolved in 2 × sodium dodecyl sulfate loading buffer, boiled for 10 min and electrophoresed after an ice bath and centrifugation. Subsequently, the proteins in the gel were transferred to the nitrocellulose membrane which was then sealed with 5 % skim milk powder at 4 °C overnight. Next, primary antibody PCSK6 (1:1000), NLRP3 (1:500) (both from Abcam, Cambridge, UK) were added for overnight incubation, and then secondary antibody horseradish peroxidase-labeled immunoglobulin G (1:1000, Boster Biological Technology Co., Ltd., Wuhan, China) was added for 1-h incubation at 37 °C. The membrane was immersed in electrochemiluminescence reaction solution (Pierce, Rockford, IL, USA) for 1 min, followed by observation after exposure in a dark environment, development and photographic fixing. GAPDH was used as the internal reference. Protein bands were analyzed with the ImageJ2x software.

### PE cell modeling and culture

The trophoblastic HTR-8/SVneo (ATCC, VA, USA) and HPT-8 cell lines (Shanghai Institute of Biochemistry and Cell Biology, Chinese Academy of Sciences, Shanghai, China) were cultured in RPMI 1640 medium with 10 % fetal bovine serum (FBS) (Gibico, Grand Island, USA) (37℃, 5 % CO_2_).

HTR-8/SVneo and HPT-8 cells were cultured in an anoxic three-gas incubator (Thermo Fisher Scientific, Basingstoke, UK) for 8 h (2 % O_2_, 5 % CO_2_, 93 % N_2_), and then cultured under conditions of 20 % O_2_, 5 % CO_2_, and 75 % N_2_ for 16 h (two cycles).

### Cell hypoxia/reoxygenation (H/R) modeling and grouping

HTR-8/SVneo and HPT-8 cells were categorized as follows: H/R group (HTR-8/SVneo and HPT-8 cells treated with H/R); H/R + mimic negative control (NC) group (HTR-8/SVneo and HPT-8 cells treated with H/R after transfection with miR-135 mimic-NC); H/R + miR-135 mimic group (HTR-8/SVneo and HPT-8 cells treated with H/R after transfection with miR-135 mimic); H/R + siRNA NC group (HTR-8/SVneo and HPT-8 cells treated with H/R after transfection with PCSK6 siRNA NC); H/R + PCSK6-siRNA group (HTR-8/SVneo and HPT-8 cells treated with H/R after transfection with PCSK6-siRNA); H/R + overexpression (OE) NC group (HTR-8/SVneo and HPT-8 cells treated with H/R after transfection with PCSK6 OE NC); H/R + OE-PCSK6 group (HTR-8/SVneo and HPT-8 cells treated with H/R after transfection with OE-PCSK6); H/R + OE-PCSK6 + MCC950 group (HTR-8/SVneo and HPT-8 cells treated with H/R after transfection with OE-PCSK6 and incubation with 0.01 μm MCC950 [NLRP3 inhibitor] for 1 h). Normal HTR-8/SVneo and HPT-8 cells with no treatment were set as a control group. The above oligonucleotides and plasmids were purchased from Guangzhou RiboBio Co., Ltd. (Guangzhou, China). The cells were seeded in a six-well plate 24 h before transfection. When the cell confluence reached 50 %, HTR-8/SVneo and HPT-8 cells were transiently transfected with lipofectamine2000 (Invitrogen, California, USA). After 6 h, the transfection in each well was replaced with 2 mL RPMI 1640 medium with 10 % FBS.

### 3-(4,5-dimethylthiazol-2-yl)-2,5-diphenyltetrazolium bromide (MTT) assay

Cells were inoculated in a 96-well plate at 4 × 10^3^ cells/well and cultured for 6, 24, 48, 72 and 96 h, respectively, and 3 replicate wells were set up at each time point. Twenty µL of 5 g/mL MTT (Sigma-Aldrich, St. Louis, MO, USA) solution was added to each well, and after 4-h incubation, the medium was discarded. Then 150 µL dimethyl sulfoxide was added to each well for 10 min. OD value was measured at 490 nm with a microplate reader.

### Colony formation assay

Each group of cells was detached with trypsin, and a single cell suspension was prepared by trituration. The cell counting plate was adopted to count the number of living cells, and the cell density was adjusted. Then the cells were inoculated in 6-well plates at 1000 cells/well and cultured at 37 °C with 5 % CO_2_. When colonies in the wells of the control group were visible to the naked eye after about 10 d, the culture was terminated. Then each well was supplemented with methanol for 15-min fixation, and crystal violet for 15-min staining. The number of cell colonies was counted with the naked eye by inverting the dish. Three duplicate wells were set up in each group.

### Flow cytometry

The binding buffer was diluted with deionized water at 1:3, and the cells were suspended with 250 µL binding buffer and adjusted to a concentration of 1 × 10^6^ cells/mL. Then 100 µL cell suspension was added to a 5 mL flow tube, and supplemented with 5 µL Annexin V-APC and 5 µL PI solution (BD Biosciences, New Jersey, USA) for 15-min incubation in the dark. Lastly, the samples were added to the flow cytometer after mixed with 400 µL phosphate buffered saline (PBS), and the results were analyzed by the computer.

### Transwell assay

*Invasion assay* Transwell chambers (Corning, New York, USA) were placed in a 24-well plate, and 300 µL pre-heated serum-free medium was added to the apical chambers and kept at room temperature for 30 min to rehydrate the Matrigel. Then cells in logarithmic phase were starved and cultured in serum-free medium for 24 h, and then supplemented with different media to make cell suspension after centrifugation, with the final concentration adjusted to 1 × 10^5^ cells/mL. Next, 500 µL RPMI 1640 medium with 10 % FBS was added to the culture plate, and 200 µL cell suspension from each group was inoculated in Transwell chambers and incubated at 37 °C with 5 % CO_2_. After 6-h incubation, the medium was removed. The cells in the matrigel and chambers that did not penetrate the basement membrane were wiped off with cotton swabs. Subsequently, the chambers were placed in the wells in an empty 24-well plate supplemented with 500 µL colorant for 20 min. Finally, five fields with even distribution of cells were selected under the microscope for photographing and counting.

*Migration assay* Each group of cells was trypsinized and resuspended in RPMI 1640 medium with 1 % FBS, with cell density adjusted to 1 × 10^5^ cells/well. Then 300 µL cell suspension was added to each well of the apical Transwell chambers, and 500 µL RPMI 1640 medium with 10 % FBS was added to each well of the basolateral chambers. After 48-h culture, the cells in the apical chamber were wiped off with a cotton swab. Next, 800 µL methanol was added for 15-min fixation, and crystal violet for 15-min staining. Five fields at high magnification were randomly selected under an inverted microscope (× 200), and the number of cells passing through the membrane was counted and averaged. Two duplicate wells were set up in each group.

### Dual luciferase reporter gene assay

The targeting relationship of miR-135 and PCSK6 and the binding site of miR-135 and PCSK6 3′-untranslated regions (3′-UTR) were predicted with a bioinformatics software (https://cm.jefferson.edu/rna22/Precomputed/). The PCSK6 3′-UTR promoter sequence containing the miR-135 binding site was synthesized and inserted into pMIR-REPORT™ Luciferase vector plasmid (Ambion, Austin, TX, USA) to construct PCSK6 3′-UTR wild type plasmid (PCSK6-WT), based on which, the binding site was mutated for PCSK6 3’-UTR mutant type plasmid (PCSK6-MUT) establishment on the basis of the procedures of plasmid extraction kit (Promega, Madison, Wisconsin, USA). Then HTR-8/SVneo and HPT-8 cells in logarithmic phase were inoculated in the 96-well plate, and transfected with the mixtures of PCSK6-WT and PCSK6-MUT plasmids with mimic NC and miR-135 mimic respectively, by Lipofectamine 2000 at about 70 % cell density. After 48 h, cells were lysed for luciferase activity detection with a luciferase assay kit (BioVision, San Francisco, CA, USA) by Glomax20/20 luminometer (Promega, Madison, Wisconsin, USA). Each experiment was repeated three times.

### Statistical analysis

All data were processed with SPSS 21.0 statistical software (IBM Corp., Armonk, NY, USA). The measurement data were expressed as mean ± standard deviation. The comparison between two groups was performed with independent sample t-test, and that among multiple groups with one-way analysis of variance (ANOVA), after which pairwise comparison was made by Tukey’s multiple comparison test. *P <* 0.05 indicated statistically significant difference.

## Results

### Clinical data of PE patients

The clinical data of the patient was shown in Table 2. There were no significant differences in age, gestational age, and body mass index between normal pregnant women and those with PE. Mean arterial pressure, systolic blood pressure and diastolic blood pressure and urine protein while placental weight decreased in PE patients (all *P* < 0.05).

**Table 2:** Clinical information of the patients

Clinical date	Control (n = 25)	PE (n = 25)	P
Maternal age (years)	26.73 ± 4.34	28.91 ± 5.42	0.123
Gestational age (weeks)	37.31 ± 0.59	37.03 ± 0.92	0.2064
BMI (kg/m^2^)	25.15 ± 1.58	24.69 ± 1.67	0.3221
MABP (mmHg	96.87 ± 3.34	121.47 ± 6.13	< 0.0001
SBP (mmHg)	107.52 ± 12.61	164.25 ± 13.82	< 0.0001
DBP (mmHg)	77.92 ± 6.68	106.33 ± 11.94	< 0.0001
Proteinuria (g/24 h)	N/A	3.92 ± 0.63	N/A
Neonatal weight (g)	3285.68 ± 183.58	2789.24 ± 204.65	< 0.0001
PLW (g)	667.53 ± 46.75	478.27 ± 37.66	< 0.0001

*BMI* body mass index, *MABP* mean arterial blood pressure, *SBP* systolic blood pressure, *DBP* diastolic blood pressure, *PLW* placental weight, *PE* preeclampsia

### MiR-135 is down-regulated and PCSK6 and NLRP3 are up-regulated in placenta tissues of PE patients

**Fig. 1:**
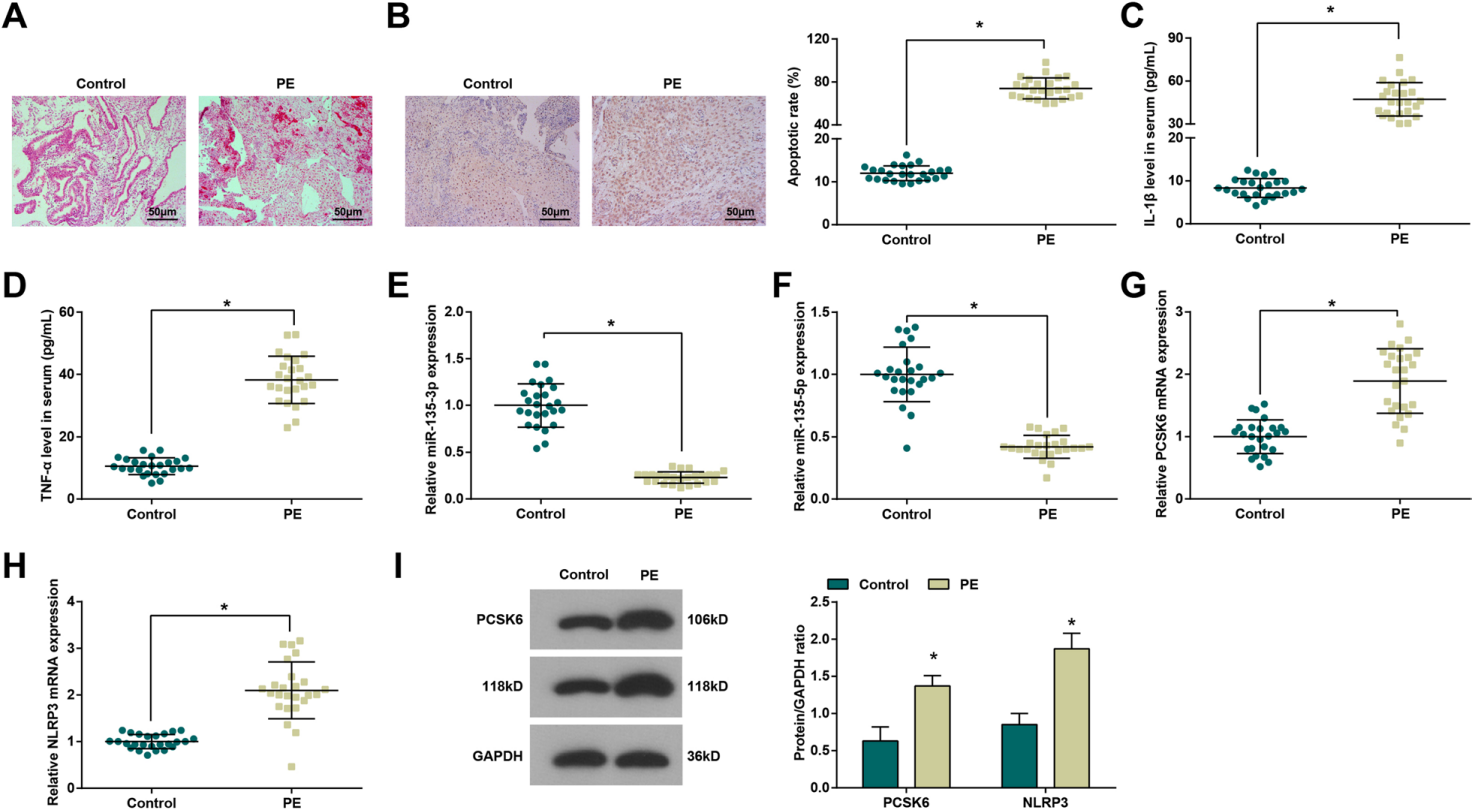
MiR-135 is down-regulated and PCSK6 and NLRP3 is up-regulated in placenta tissues of PE patients. **A** HE staining of clinical placenta tissues (× 200); **B** TUNEL staining of clinical placenta tissues (× 200); **C**, **D** Detection of IL-1β and TNF-α levels in serum by ELISA; **E** Detection of miR-135-3p expression by qPCR; F. Detection of miR-135-5p expression by qPCR; **G** Detection of PCSK6 expression by qPCR; **H** Detection of NLRP3 expression by qPCR; **I** Detection of PCSK6 and NLRP3 protein expression by Western blot analysis; n = 25. *P < 0.05 vs. the control group

In addition to that, we also conducted HE staining (Fig. 1A) with placenta tissues and observed that placenta tissues in the control group showed mature villi, uniform matrix, full morphology, less mesenchyme, abundant blood vessels, and orderly arranged nuclei of syncytiotrophoblast cells. Placenta tissues in the PE group suggested immature villi, reduced villus vessels, and edematous mesenchyme, disorderly arranged nuclei of syncytiotrophoblast cells, increased cellulose-like necrosis around the villi, decreased blood vessels, and narrow lumen. TUNEL staining showed that the nuclei of apoptotic cells were yellow or brownish yellow, and the nuclei showed different degrees of pyknosis and different shapes (round, lobulated or irregular). Apoptotic rate of the trophoblasts in the PE group suggested a visible rise versus the control group (*P <* 0.05; Fig. 1B).

ELISA results indicated elevation of IL-1β and TNF-α levels in the PE group in relation to the control group (both *P <* 0.05; Fig. 1C, D).

qPCR demonstrated versus the control group, miR-135 expression was lower in the PE group (*P <* 0.05), and miR-135-3p expression was lower than that of miR-135-5p (Fig. 1E, F), suggesting that the up-regulating miR-135-3p may have a better therapeutic effect than up-regulating miR-135-5p in PE trophoblasts. Thus, we further explored the role of miR-135-3p. QPCR and Western blot analysis fond that PCSK6 and NLRP3 mRNA and protein expression increased in the PE group by contrast to the control group (both *P <* 0.05; Fig. 1G–I).

### Establishment of H/R trophoblasts model

**Fig. 2:**
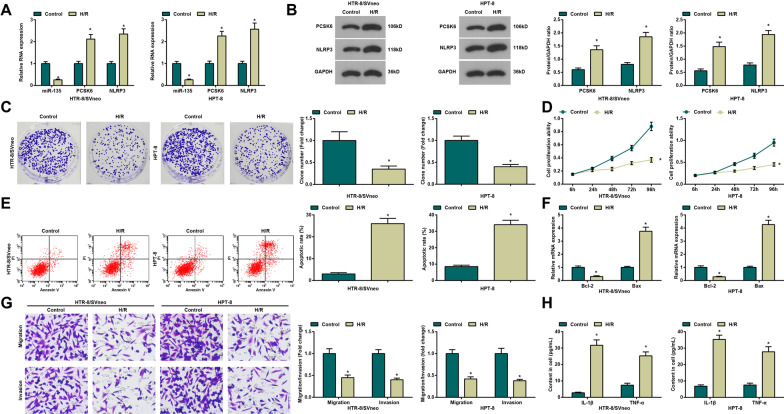
Establishment of H/R trophoblasts model. **A** Detection of miR-135, PCSK5 and NLRP3 expression in H/R-treated HTR-8/SVneo and HPT-8 trophoblasts by qPCR; **B** Detection of PCSK5 and NLRP3 protein expression in H/R-treated HTR-8/SVneo and HPT-8 trophoblasts by Western blot; **C** Detection of cell proliferation in H/R-treated HTR-8/SVneo and HPT-8 trophoblasts by colony formation assay; **D** Detection of cell viability in H/R-treated HTR-8/SVneo and HPT-8 trophoblasts by MTT assay; **E** Detection of cell apoptosis in H/R-treated HTR-8/SVneo and HPT-8 trophoblasts by flow cytometry; **F** Detection of Bcl-2 and Bax mRNA expression in H/R-treated HTR-8/SVneo and HPT-8 trophoblasts by qPCR; **G** Detection of invasion and migration in H/R-treated HTR-8/SVneo and HPT-8 trophoblasts by Transwell test; **H** Detection of IL-1β and TNF-α expression in H/R-treated HTR-8/SVneo and HPT-8 trophoblasts by ELISA; N = 3, *P < 0.05 vs. the control group

qPCR and Western blot analysis were adopted for miR-135, PCSK6 and NLRP3 expression detection in HTR-8/SVneo and HPT-8 cells (Fig. 2A, B). MiR-135 expression dropped and PCSK6 and NLRP3 expression rose in the H/R group relative to the control group (all *P <* 0.05).

The result of colony formation assay and MTT assay (Fig. 2C, D), flow cytometry (Fig. 2E), qPCR (Fig. 2F), Transwell (Fig. 2G) and ELISA (Fig. 2H) revealed that the proliferation ability and viability, invasion and migration, in concert with Bcl-2 mRNA expression showed a great reduction while inflammation, apoptosis rate and Bax mRNA expression manifested an increase in the PE group versus the control group (all *P <* 0.05). Thus, it was confirmed that H/R successfully mimics PE in cells.

### Up-regulating miR-135 alleviates inflammatory response in H/R-treated trophoblasts

**Fig. 3:**
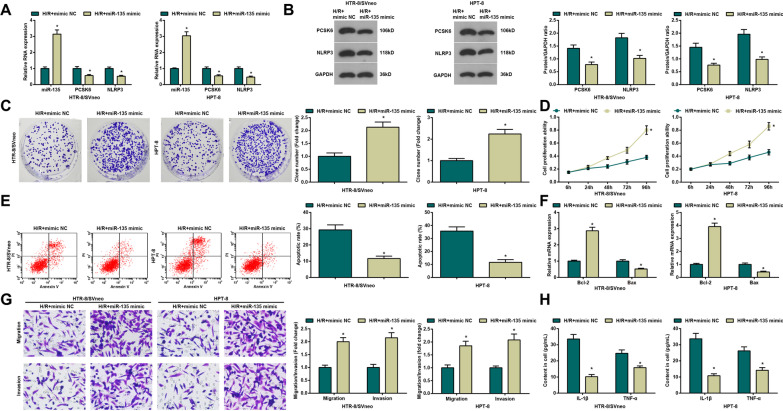
Up-regulating miR-135 alleviates inflammatory response in H/R-treated trophoblasts. **A** Detection of miR-135, PCSK5 and NLRP3 expression in H/R-treated HTR-8/SVneo and HPT-8 trophoblasts by qPCR; **B** Detection of PCSK5 and NLRP3 protein expression in H/R-treated HTR-8/SVneo and HPT-8 trophoblasts by Western blot; **C** Detection of cell proliferation in H/R-treated HTR-8/SVneo and HPT-8 trophoblasts by colony formation assay; **D** Detection of cell viability in H/R-treated HTR-8/SVneo and HPT-8 trophoblasts by MTT assay; **E** Detection of cell apoptosis in H/R-treated HTR-8/SVneo and HPT-8 trophoblasts by flow cytometry; **F** Detection of Bcl-2 and Bax mRNA expression in H/R-treated HTR-8/SVneo and HPT-8 trophoblasts by qPCR; **G** Detection of invasion and migration in H/R-treated HTR-8/SVneo and HPT-8 trophoblasts by Transwell test; **H** Detection of IL-1β and TNF-α expression in H/R-treated HTR-8/SVneo and HPT-8 trophoblasts by ELISA; N = 3, *P < 0.05 vs. the H/R + mimic NC group

To explore the role of miR-135 in PE, we applied miR-135 mimic to transfect H/R-treated cells (HTR-8/SVneo and HPT-8 cells). The results demonstrated that miR-135 expression enhanced while PCSK6 and NLRP3 expression decreased in the H/R + miR-135 mimic group versus the H/R + mimic NC group (all *P <* 0.05) (Fig. 3A, B).

Then, cell proliferation (Fig. 3C, D), invasion and migration (Fig. 3G), Bcl-2 mRNA expression (Fig. 3F) were found to increase while apoptosis rate (Fig. 3E), Bax mRNA expression (Fig. 3F), and inflammatory indices (IL-1β and TNF-α) (Fig. 3H) to decrease in the H/R + miR-135 mimic group versus the H/R + mimic NC group (all *P <* 0.05).

In summary, up-regulating miR-135 alleviated inflammatory response in H/R-treated cells.

### MiR-135 targets PCSK6

**Fig. 4:**
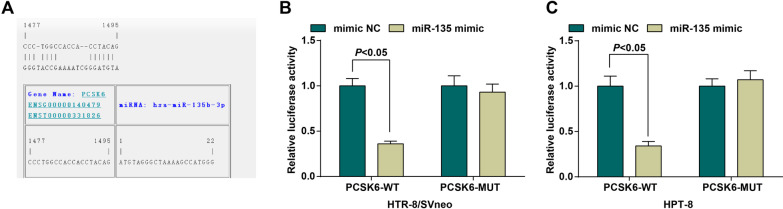
MiR-135 targets PCSK6. **A** Prediction of the binding site between miR-135 and PCSK6’ **B**, **C** Detection of luciferase activity of HTR-8/SVneo and HPT-8 cells by dual luciferase reporter gene assay; N = 3

Online analysis software revealed that there was a specific binding site between PCSK6 gene sequence and miR-135 sequence, and PCSK6 was miR-135’s target gene (Fig. 4A). The luciferase reporter assay was adopted to verify that miR-135 targeted PCSK6 in HTR-8/SVneo and HPT-8 cells (Fig. 4B, C). The results illustrated that the luciferase activity of both kinds of cells cotransfected with WT-miR-135/PCSK6 descended (*P <* 0.05), while that of cells cotransfected with MUT-miR-135/PCSK6 had no notable difference (*P >* 0.05), indicating that miR-135 can specifically bind to PCSK6.

### Depletion of PCSK6 enhances growth of H/R-treated trophoblasts

**Fig. 5:**
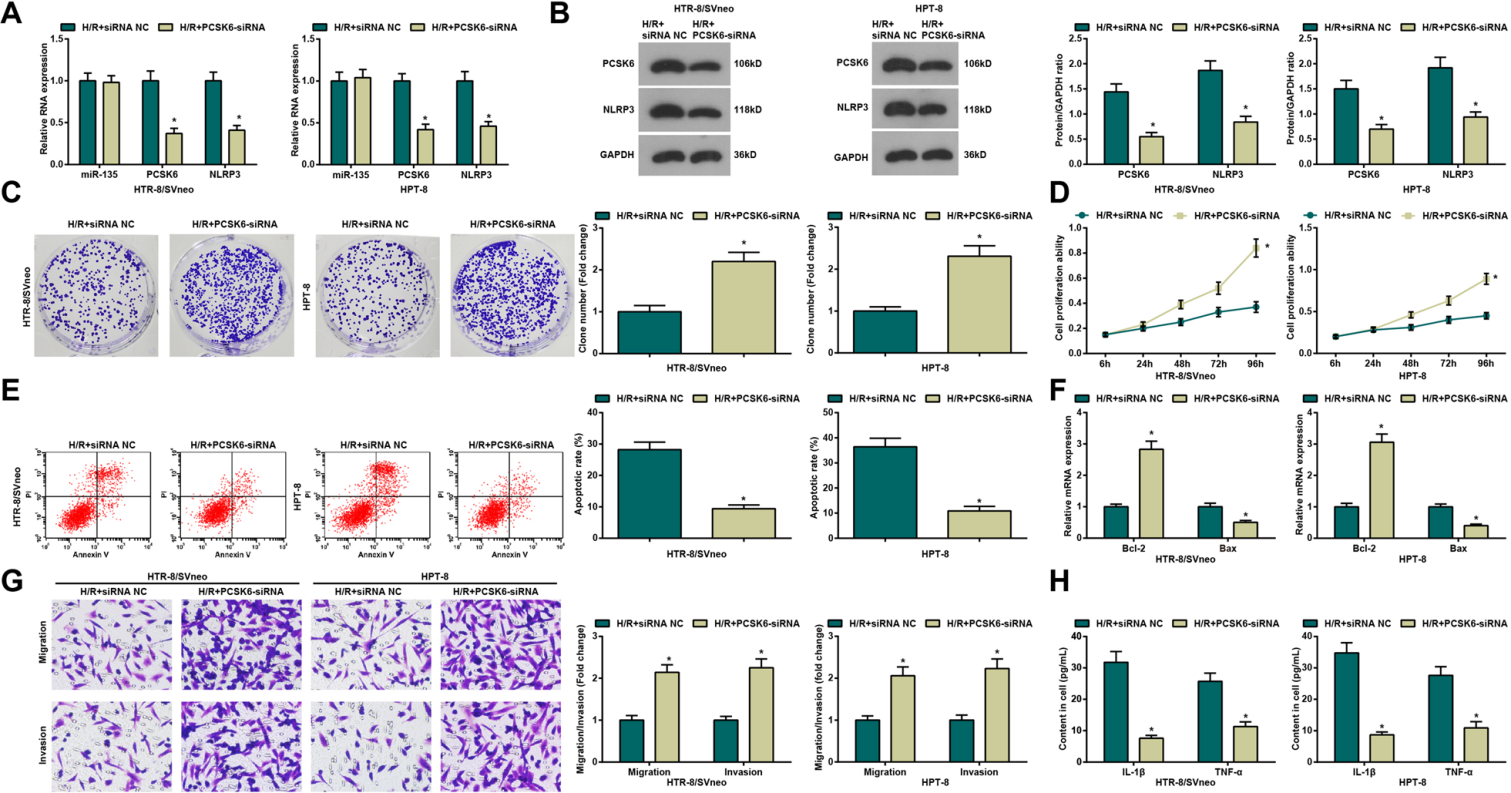
Depletion of PCSK6 enhances growth of H/R-treated trophoblasts. **A** Detection of miR-135, PCSK5 and NLRP3 expression in H/R-treated HTR-8/SVneo and HPT-8 trophoblasts by qPCR; **B** Detection of PCSK5 and NLRP3 protein expression in H/R-treated HTR-8/SVneo and HPT-8 trophoblasts by Western blot; **C** Detection of cell proliferation in H/R-treated HTR-8/SVneo and HPT-8 trophoblasts by colony formation assay; **D** Detection of cell viability in H/R-treated HTR-8/SVneo and HPT-8 trophoblasts by MTT assay; **E** Detection of cell apoptosis in H/R-treated HTR-8/SVneo and HPT-8 trophoblasts by flow cytometry; **F** Detection of Bcl-2 and Bax mRNA expression in H/R-treated HTR-8/SVneo and HPT-8 trophoblasts by qPCR; **G** Detection of invasion and migration in H/R-treated HTR-8/SVneo and HPT-8 trophoblasts by Transwell test; **H** Detection of IL-1β and TNF-α expression in H/R-treated HTR-8/SVneo and HPT-8 trophoblasts by ELISA; N = 3, *P < 0.05 vs. the H/R + siRNA NC group

Next, the mechanism of PCSK6 in PE was uncovered through transfection with siRNA-PCSK6 into HTR-8/SVneo and HPT-8 cells. Then, we observed that PCSK6 and NLRP expression reduced in the H/R + PCSK6-siRNA group with respect to the H/R + siRNA NC group (all *P <* 0.05) (Fig. 5A, B). Subsequently, we further detected higher proliferation, migration and invasion, and Bcl-2 mRNA expression, as well as lower apoptosis rate, inflammation and Bax mRNA expression in the H/R + PCSK6-siRNA group than the H/R + siRNA NC group (all *P <* 0.05) (Fig. 5C–H).

In conclusion, silencing PCSK6 enhances growth of H/R-treated HTR-8/SVneo and HPT-8 cells.

### Inhibition of NLRP3 relieves the inflammatory cascade of HTR8/SVneo cells after H/R

**Fig. 6:**
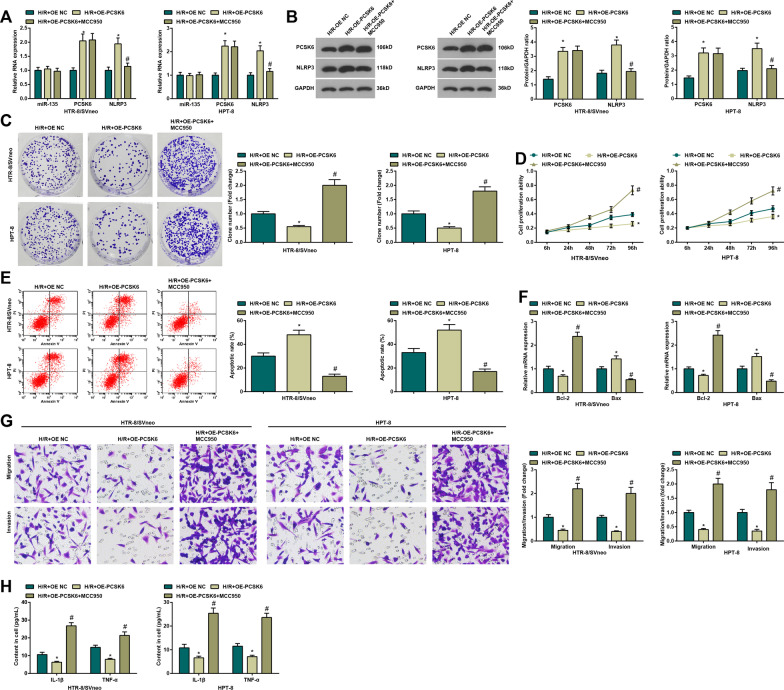
Inhibition of NLRP3 relieves the inflammatory cascade of HTR8/SVneo cells after H/R. **A** Detection of miR-135, PCSK5 and NLRP3 expression in H/R-treated HTR-8/SVneo and HPT-8 trophoblasts by qPCR; **B** Detection of PCSK5 and NLRP3 protein expression in H/R-treated HTR-8/SVneo and HPT-8 trophoblasts by Western blot; **C** Detection of cell proliferation in H/R-treated HTR-8/SVneo and HPT-8 trophoblasts by colony formation assay; **D** Detection of cell viability in H/R-treated HTR-8/SVneo and HPT-8 trophoblasts by MTT assay; **E** Detection of cell apoptosis in H/R-treated HTR-8/SVneo and HPT-8 trophoblasts by flow cytometry; **F** Detection of Bcl-2 and Bax mRNA expression in H/R-treated HTR-8/SVneo and HPT-8 trophoblasts by qPCR; **G** Detection of invasion and migration in H/R-treated HTR-8/SVneo and HPT-8 trophoblasts by Transwell test; (H) Detection of IL-1β and TNF-α expression in H/R-treated HTR-8/SVneo and HPT-8 trophoblasts by ELISA; N = 3, *P < 0.05 vs. the H/R + OE NC group; ^#^*P* < 0.05 vs. the H/R + OE-PCSK6 group

Finally, the combined role of PCSK6 and NLRP3 in PE was further investigated. Versus the H/R + OE NC group, the H/R + OE-PCSK6 group showed increased PCSK6 and NLRP3 expression while the H/R + OE-PCSK6 + MCC950 group exhibited reduced NLRP3 with comparison to the H/R + OE-PCSK6 group (all *P <* 0.05) (Fig. 6A, B). Furthermore, we recognized that proliferation, migration and invasion, and Bcl-2 mRNA expression were suppressed, whilst apoptosis rate, inflammation and Bax mRNA expression were promoted in the H/R + OE-PCSK6 group with respect to the H/R + OE NC group (all *P <* 0.05); in contrast to the H/R + OE-PCSK6 group, the H/R + OE-PCSK6 + MCC950 group demonstrated promotion of cell growth, and reduction of inflammatory response (all *P <* 0.05) (Fig. 6C–H). In short, inhibition of NLRP3 relieves the inflammatory cascade of HTR8/SVneo cells after H/R.

## Discussion

PE is still a prominent problem for mothers and babies around the world with considerable morbidity (Myatt et al. 2014). The challenge of opportune and correct recognition and management of PE often result from nonspecific signs and symptoms at diagnosis and the poor correlation of common severity criteria with adverse maternal and fetal outcomes (Herraiz et al. 2018). Lately, miRNAs with altered expression have been reported to be concerned with PE development (Yang et al. 2015). In this present study, we discuss the mechanism of miR-135 on the inflammatory response of PE via modulation of PCSK6/NLRP3. Taken together, we revealed that miR-135 is down-regulated while PCSK6 and NLRP3 are up-regulated in PE, and enhancement of miR-135 or reduction of PCSK6 attenuates inflammatory response in PE by controlling NLRP3 inflammasome expression.

Initially, qPCR and Western blot analysis revealed that miR-135 was down-regulated while PCSK6 and NLRP3 inflammasome were up-regulated in placenta tissues in PE patients and PE models of HTR-8/SVneo and HPT-8 cells. Also, we got another finding that miR-135 targets PCSK6 through dual luciferase reporter gene assay. In compliance with our results, there is literature showing that miR-145 expression is declined in placental tissues in PE, which is similar to the finding in a study that miR-29a/c-3p expression is reduced in unpassaged human umbilical vein endothelial cells in PE patients (Han et al. 2017; Zhou et al. 2017). It is reported that miR-135 is down-regulated in MPP^+^-intoxicated SH-SY5Y and breast cancer cells (Jiang et al. 2019; Zhang et al. 2017a, b). Some researchers have proffered that PCSK6 is miR-124’s target gene and PCSK6 gene expression is inversely related to miR-124 expression (Kang et al. 2014). Overexpressed PCSK6 has been proposed in pancreatic cancer in a previous study, and Lin YE et al. have also verified that PCSK6 is enhanced in non-small cell lung cancer (Tian et al. 2016; Lin et al. 2015). Evidence has indicated that miR-233 overexpression abates NLRP3 expression (Neudecker et al. 2017). It is also demonstrated that NLRP3 inflammasome expression is enhanced in ischemia-like conditions like stroke patients (Fann et al. 2013).

Furthermore, we illustrated that miR-135 enhancement and PCSK6 silencing strengthened colony formation ability, viability, and invasion and migration ability, elevated Bcl-2 levels, and weakened apoptosis, Bax, IL-1β and TNF-α levels of HTR-8/SVneo and HPT-8 cells. At the same time, we discovered that overexpression of PCSK6 reverses the impacts of miR-135 enhancement on PE progression. Similar to our findings, there is study reporting that miR-144 expression was lessened in PE, and HTR8/SVneo cells transfected with miR-144 mimic present prominent increase in proliferation, migration and invasion, and abatement in apoptosis (Xiao et al. 2017). It has been indicated that miR-195 shows low expression in PE, and aberrantly expressed miR-195 is likely to boost the occurrence of PE by modulating ActRIIA-mediated Activin/Nodal signaling in human placenta (Bai et al. 2012). In fact, enhanced miR-135-5p could negatively regulate the features of allergic inflammation (Kim et al. 2019). Also, overexpressing miR-135b has the great ability to enhance proliferation and reduce apoptosis and inflammatory release of MPP^+^-intoxicated neurons (Zhang et al. 2017a, b). A previous report has suggested that restriction of PCSK6 protects rat models of rheumatoid arthritis against synovitis (Jiang et al. 2015). Similarly, a recent study has shown that limitation of PCSK6 impedes prostate cancer growth in an androgen-independent manner (Couture et al. 2017). Also, there is research revealing that loss of PCSK6 increases outward remodeling and reduces medial contractility in mice (Kim et al. 2019). till now, very little study has mentioned the interaction between miR-135 and PCSK6 with NLRP3, which still requires much more explorations.

## Conclusions

All in all, we demonstrated down-regulated miR-135 and up-regulated PCSK6 and NLRP3 in PE, and confirmed that enhancement of miR-135 and reduction of PCSK6 abate inflammatory response in PE through restriction of NLRP3 inflammasome expression. Exploration of miR-135 in PE development yields a better knowledge of the role of miR-135/PCSK6/NLRP3 axis in PE and confirmed it a new biomarker in PE progression and treatment. Nevertheless, in this study, large-scale studies are expected to be conducted to further elucidate the exact role of miR-135 in the inflammatory response of PE, and this study is essential to understand the mechanisms involved in miR.

## Data Availability

Not applicable.

## Abbreviations

miRNAs: microRNAs
PE: Preeclampsia
PCSK6: Proprotein convertase subtilisin/kexin-6
NLRP3: NLR pyrin domain containing 3
HE: Hematoxylin-eosin
TEM: Transmission electron microscope
ELISA: Enzyme linked immunosorbent assay
OD: Optical density
TDT: Terminal deoxyribonucleotidyl transferase
qPCR: Quantitative polymerase chain reaction
GAPDH: Glyceraldehyde-3-phosphate dehydrogenase
PCNA: Proliferating cell nuclear antigen
MMP: Matrix metalloproteinase
FBS: Fetal bovine serum
OE: Overexpression
PI: Propidium iodide
3′-UTR: 3′-untranslated regions
ANOVA: Analysis of variance
